# The effect of nudges on autonomy in hypothetical and real life settings

**DOI:** 10.1371/journal.pone.0256124

**Published:** 2021-08-24

**Authors:** Jonas Wachner, Marieke A. Adriaanse, Denise T. D. De Ridder

**Affiliations:** Social-, Health-, and Organizational Psychology, Utrecht University, Utrecht, The Netherlands; Middlesex University, UNITED KINGDOM

## Abstract

Nudges have repeatedly been found to be effective, however they are claimed to harm autonomy, and it has been found that laypeople expect this too. To test whether these expectations translate to actual harm to experienced autonomy, three online studies were conducted. The paradigm used in all studies was that participants were asked to voluntarily participate in a longer version of the questionnaire. This was either done in a hypothetical setting, where participants imagined they were asked this question, but did not answer it, and reported their expectations for autonomy; Or in an actual choice setting where participants answered the question and then reported their actual autonomy. The first study utilized the hypothetical setting and tried to replicate that laypeople expect nudges to harm autonomy with the current paradigm. A total of 451 participants were randomly assigned to either a control, a default nudge, or a social norm nudge condition. In the default nudge condition, the affirmative answer was pre-selected, and in the social norm nudge condition it was stated that most people answered affirmative. The results showed a trend for lower expected autonomy in nudge conditions, but did not find significant evidence. In Study 2, with a sample size of 454, the same design was used in an actual choice setting. Only the default nudge was found to be effective, and no difference in autonomy was found. In Study 3, Studies 1 and 2 were replicated. Explanation of the nudge was added as an independent variable and the social norm nudge condition was dropped, resulting in six conditions and 1322 participants. The results showed that participants indeed expected default nudges to harm their autonomy, but only if the nudge was explained. When actually nudged, no effect on autonomy was found, independent of the presence of an explanation.

## Introduction

Nudges are subtle changes in the way options are presented, designed to influence decisions in a predictable way [1] and achieved by relying on well-known decision-making tendencies. Monetary incentives for a specific option, or making certain options impossible, do not qualify as nudges [1]; by contrast, placing fruit near a cash register to make this nutritional option more salient does count as a nudge. Rather than prohibiting unhealthy choices or making the unhealthy option more expensive, placing the desired option more prominently among less desired options increases customers’ focus on the healthy options by making them more easily accessible [2].

Different types of nudges have proven effective across various settings. Placing fruit items next to a cash register at a kiosk increases the number of healthy snacks sold [3]. If enrollment in a retirement saving plan is the default option, employee enrollment is significantly higher [4], and informing people of how many ecological products the average customer buys does increase the number of such products sold [5]. Besides proving effective, nudges are inexpensive and easy-to-implement interventions that change behaviors and decisions [6]. However, nudges have also been criticized. One prominent criticism is that nudges may harm people’s autonomy, as decision makers are unable to protect themselves against the influence of subtle nudges—influence of which they are often unaware [7, 8]. Given autonomy’s crucial role for physical and mental well-being [9, 10], this would be concerning if proved correct. Indeed, a recent study found that participants *expect* nudges to harm their autonomy [11].

In contrast, another study found that scenarios which included a nudge by a health professional were not expected to be harmful to autonomy [12]. However, in this study participants were not being nudged themselves, but rather asked to rate the scenario from a third person viewpoint. Earlier research has demonstrated that in the case of nudging there is a critical difference in how people evaluate choice support for themselves as compared to choice support for others: they tend to view support for other people as less problematic and even required, while they consider support in making their own decisions as unwanted interference [13], illustrating that expectations for one’s own autonomy are not identical to expectations about other people’s autonomy.

Ultimately, the criticism argues that nudges harm the *experience* of autonomy after being nudged, while the beforementioned studies only investigated laypeople’s *expected* autonomy. In the current paper, we will argue that these two concepts should be seen as distinct, with each having its own merit. The current paper aims to replicate previous findings regarding *expected* autonomy and investigates *experienced* autonomy in a similar setting. Thereby, we will test the claims outlined above, as well as enhance understanding of the relationship between autonomy expectations and experiences.

### Nudges and autonomy

Whereas several meta-analyses suggest that nudges effectively steer behavior in a desired direction [14–16], opponents of nudges have argued that the use of nudging may thwart people’s autonomy. These arguments are rooted in the notion that autonomy is equivalent with conscious free choice upon which people are able to reflect. Although other conceptions of autonomy are being used in philosophical reflection on nudging, such as offering people the chance to act in line with their intentions (see for an overview [17]), the most pervasive criticism of nudges potentially harming autonomy relates to the idea that free, unguided choice is crucial to autonomy. The concerns are twofold.

First, philosophers have argued that nudges influence decisions without the decision maker being aware of these influences; this hinders decision makers’ opportunity to make an autonomous choice [7, 8]. This concern might be resolved by the use of transparent nudges, i.e., nudges accompanied by an explicit explanation of their purpose and their working mechanism, which have been proven as effective as non-transparent nudges [18–20]. The second autonomy concern is, however, not resolved by the deployment of transparent nudges. The effectiveness of nudges relies on mechanisms through which nudges influence decisions (e.g., proximity, salience, defaults, anchoring) that are deemed not meaningful, by decision makers themselves, in the process of making decisions. For that reason, Wilkinson [21] has argued that a decision process is ‘perverted’ when it is based on factors that the decision maker would normally not see as relevant to their decision.

### The importance of autonomy in nudging

Besides the ethical argument for autonomy, there are also psychological and behavioral arguments that speak to the importance of autonomous decisions. Much empirical evidence from the psychological literature illustrates the importance of autonomy for well-being. Self-Determination Theory posits that autonomy is a basic need (next to competence and relatedness [22]). It is an essential predictor of both physical and mental well-being [9, 10], and this has been empirically demonstrated in diverse settings and across different groups [23–25].

Next to its prominent role in health and well-being, autonomy is also important from a psychological perspective because it bears implications for subsequent choices. That is, research has shown that autonomous choice generally influences whether the decision maker will make a similar decision in the future [26, 27]. Considering that nudges are frequently used for guiding repeated decisions, which in and of themselves as isolated choices have little impact (e.g., decisions related to recycling, healthy eating, exercising), negative influences of nudged decisions on subsequent related decisions should be avoided [28]. This further underscores the importance of ensuring that people who follow the nudge also feel autonomous and satisfied after having made a decision, not just for reasons associated with health and well-being in the moment but also to promote similar behavior in the future.

### Expectations versus reality

Although there clearly are philosophical and psychological reasons to better understand the effect of nudges on post-choice autonomy, few studies have empirically investigated this issue. In an earlier set of studies, we asked participants to read choice scenarios, including descriptions of nudges, and rate how autonomous they expected to feel if they would have made a decision in this scenario [11]. We found that people who were presented with a default nudge consistently expected to feel less autonomous compared to people who were not presented with a nudge, while those presented with a social norm nudge expected to feel similarly autonomous compared to a control condition. Studies like these, based on how people expect nudges to impact their autonomy, explain how people think about nudges in a deliberative and general way, as they would during laypeople discussions on nudges.

How people *actually feel* after having been nudged, however, might prove different from how people *expect to* feel after being nudged. A considerable amount of research suggests that people are poor predictors of emotional responses [29, 30], of the likelihood that specific events will occur [30], and of their own reactions to future events [31]. For example, research on the impact bias suggests that people tend to overestimate the influence of events on their emotions and overall wellbeing [32]. Similarly, research on immune neglect (neglecting one’s ability to cope with negative events) demonstrates that people tend to overestimate the impact of negative events [33]. Based on these findings, we hypothesize that although participants may *expect* to feel slightly less autonomous when confronted with a nudge, they do not in fact *feel* less autonomous when nudged.

### The current research

In the present series of studies, we will investigate and compare people’s expectations about the effect of nudges on autonomy and nudges’ actual effect on autonomy. In Study 1, we aim to replicate our earlier findings regarding people’s expectations of autonomy upon being nudged [11], but we use a new nudge scenario that can also be employed in a subsequent study to investigate the experience of autonomy after actually being nudged. In Study 2, people will actually be nudged, with the nudge from the scenario in Study 1, to investigate how nudges affect experienced autonomy. In Study 3, we replicate Studies 1 and 2 simultaneously to promote an optimal comparison between the effects of nudges in hypothetical and actual nudges settings. Finally, in Study 3 we test the influence of transparency by including conditions where the nudge is made explicit and conditions where the nudge is not made explicit to investigate whether awareness of the nudge impacts autonomy. In addition, we will measure people’s satisfaction with their choice as the second primary dependent variable. Secondary dependent measures are decision making competence, experienced pressure to choose the long version of the questionnaire, how carefully participants answered the nudged question, how much participants doubted that the question will actually affect the duration of the study, and how accepting the participants were of the use of such a nudge. Materials and results for these secondary measures can all be found in the S1 File.

## Study 1

All studies were approved by the Ethics Committee of the Faculty of Social and Behavioural Sciences of Utrecht University. The approval is based on the documents send by the researchers as requested in the form of the Ethics committee and filed under number 20–150. Written consent was given by participants.

### Participants

We recruited 451 participants (47% female, mean age 30, *SD* = 10.48 [range 18–71]) through the online service Prolific. Participation was rewarded with 0.40£ and took on average five minutes. Prolific users were only eligible to participate if they have not participated in earlier studies of this research line and were fluent in English.

### Design and procedure

This online experiment used a one-factor between-subject design, with type of nudge (default nudge / social norm nudge / none) as the independent variable and expected autonomy and satisfaction as the main dependent variables. Participants were told that they had to read a hypothetical scenario. Depending on the condition they were in, it either included or did not include a nudge. Afterwards, they were asked about their expectations of feelings of autonomy and satisfaction if they would have made a decision in that scenario.

### Hypotheses

Based on findings of a previous study [11], we hypothesized participants in the default nudge condition, but not participants in the social norm nudge condition, to score lower on expected autonomy than participants in the control condition. We expected results for decision satisfaction to be similar to results for autonomy.

### Materials

*Scenario* Before the actual scenario was presented, participants received a brief explanation and reminders that the question would be hypothetical and that the researchers were not interested in how participants would answer the question, but rather how the participants feel about the question.

Participants were then asked to read the following scenario: “Imagine, you are participating in a short, 5-minute Prolific questionnaire and come across the following question: Please indicate whether you will participate in the long version of this study (+5 minutes). You will not receive additional payment, however, you will help to improve future questionnaires.”. Subsequently, participants were given two options, the ‘Longer Version’ and the ‘Normal Version’. The presentation of this question was slightly altered, depending on the experimental condition (see Experimental Manipulation below).

As this question was programmed as an image rather than as textual information to which participants could respond, it was again emphasized that participants were not required to answer the question. Participants had to wait for 20 seconds before proceeding to the next page to allow ample time for them to read the question. After 20 seconds, an explanation of the influence type was shown on the next page (see Experimental manipulation below), and after another 20 seconds, participants were allowed to proceed to the next part of the questionnaire.

#### Experimental manipulation

The presentation of the hypothetical question was slightly different among the three experimental conditions. In the default nudge condition, the option ‘Longer Version’ was checked by default. For the social norm nudge condition, no option was checked by default, but the instruction was followed by a brief note stating, “Most people chose the longer version.” The control condition had no options checked by default and no note was added.

#### Influence explanation

After seeing the hypothetical question, participants were presented with an explanation of the implemented experimental manipulation. Participants saw one of three explanations, depending on the experimental condition. The explanations of the nudge conditions comprised of a description of the nudge (e.g., default nudge: “…one option was already selected…”); the aim of the nudge (“…to increase the chance this option will be chosen.”); the mechanism by which the nudge works (e.g., social norm nudge: “By telling people that other people chose this option, it appears to be the norm…”); and finally, that this technique is called nudging and that people are usually not aware of it. Participants in the control condition read the following explanation: “As you may notice, the options are presented in a neutral manner. This is done so that the way in which the question is presented does not influence your decision.”

#### Autonomy questionnaire

Before filling in the autonomy and satisfaction questionnaires, participants were informed that ‘we will ask you about how you think you would have felt, had you actually made a decision in the described scenario’. Participants’ expected autonomy was assessed by the autonomy subscale of the Basic Psychological Needs in Exercise Scale (BPNES; [34]), which in its original form measures autonomy in a physical exercise context but was adjusted for this study to assess autonomy in a decision-making context (see Appendix). It comprises four statements (e.g., “I feel that my choice is definitely an expression of myself.”), which participants rated on five-point scales (“strongly disagree” to “strongly agree”). The four scores were averaged into one expected autonomy score (ranging from 1 to 5) with acceptable reliability (Cronbach’s α = .75).

#### Choice satisfaction questionnaire

Participants’ expected satisfaction with their choice was measured with the Decision Regret Scale [35], consisting of five statements (e.g., “It was the right decision.”), which participants rated on a five-point scale (“strongly disagree” to “strongly agree”; See S1 Appendix). The five scores were averaged to one satisfaction score with acceptable reliability (Cronbach’s α = .77).

### Results

#### Descriptives

Participants reported relatively high expectations for autonomy (*M* = 3.85, *SD* = 0.67) and satisfaction (*M* = 4.02, *SD* = 0.61). Autonomy and satisfaction were strongly correlated (*r* = .59, *p* < .01).

#### Randomization check

We conducted an ANOVA with condition as the independent variable and age as the dependent variable and found that randomization was successful (*p* = .620). We also conducted a Chi-squared test and found that sex was successfully randomized across the conditions (*p* = .200).

#### Autonomy and satisfaction

We conducted a MANOVA with autonomy and satisfaction as the dependent variables, and condition as the independent variable. The multivariate effect was significant Wilk’s Λ = 0.969, *F*(2, 448) = 3.60, *p* = .007. Both the effect on autonomy *F*(2, 448) = 3.06, *p* = .048 and satisfaction *F*(2, 448) = 6.29, *p* = .002 were significant.

A subsequent post-hoc Tukey HSD test was conducted for the effect of condition on autonomy, which showed no significant differences between the control (*M* = 3.96, *SD* = 0.57) and the default nudge condition (*M* = 3.80, *SD* = 0.73, *p* = .081) or between the control and the social norm nudge condition (*M* = 3.80, *SD* = 0.71, *p* = .084). Moreover, no significant difference was found between the default nudge and control condition (*p* = 1.00).

**Fig 1:**
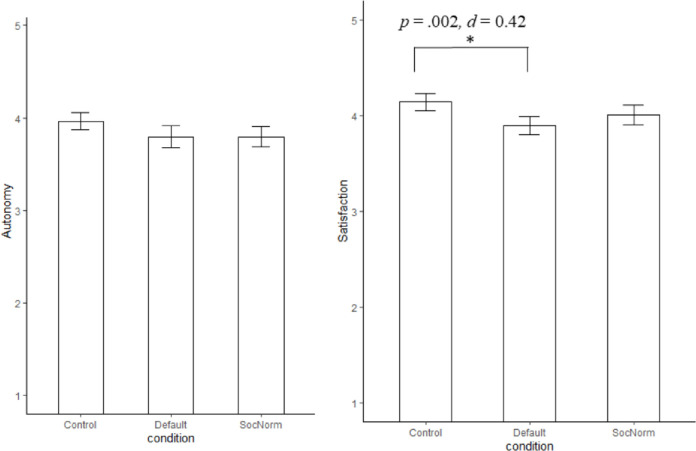
Means and standard errors for autonomy and satisfaction.

A similar post-hoc Tukey HSD test for satisfaction showed a significant, small difference between the default nudge (*M* = 3.90, *SD* = 0.59) and control condition (*M* = 4.14, *SD* = 0.57, *p* = .002, *d* = 0.42). There were no differences between the default nudge and social norm nudge condition (*M* = 4.01, *SD* = 0.63, *p* = .239) and the control and social norm nudge condition (*p* = .132; See Fig 1 for means, standard errors, and significant differences between the conditions on autonomy and satisfaction).

### Discussion

Overall, participants across all three conditions expected to feel quite autonomous and satisfied. Although the means were in the same direction as in our previous study [11], in the present study participants in the nudge conditions did not differ significantly on autonomy compared to the control condition. As hypothesized, participants who had been exposed to the default nudge, but not participants exposed to the social norm nudge, expected to be less satisfied with their choice compared to the control condition.

One reason why effects on autonomy were smaller in the present study may be the personal relevance of the choice that was nudged. While our earlier studies were about hypothetical choices that involved regular payments of money, the hypothetical choice in the current study was about the investment of an extra five minutes of time. These lowered stakes may be the reason that nudges were not seen as bigger threats to autonomy, although it is unclear to what extent the personal relevance of a free choice determines the severity of reactance [36]. In Study 2, we will present the same nudges but this time as actual nudges.

## Study 2

### Method

#### Participants

We recruited 454 participants (50% female, mean age 29, *SD* = 9.58 [range 18–70]) through the online service Prolific. Participation was rewarded with 0.40£ and the study took an average of five minutes. Prolific users were eligible to participate if they had not participated in earlier studies of the present line of research and were identified as being fluent in English.

#### Design and procedure

In contrast to Study 1, participants were asked to actually choose the long or short version of the questionnaire. Following the request, participants were asked how careful they had been in making this choice and whether they believed that their answer would actually change the survey length. Then, participants filled in the autonomy and satisfaction questionnaires. Afterwards, participants were informed that their answers had not affected the length of the questionnaire and that the aim of the experiment was to investigate the influence of the presentation of a question. Subsequently, they were thanked for their participation.

#### Hypotheses

As no comparable study had investigated experienced autonomy after a non-hypothetical nudge, we were not able to base the hypotheses on earlier findings, however, predictions can be made based on related literature. Based on the literature suggesting that people may overestimate the impact of future events [30], expectations of how people would react to nudges might not translate into experiences. Additionally, as participants in the nudge conditions will likely not be aware of the nudge, they will in turn probably not feel any threat to their autonomy and therefore have no reason to be less satisfied with their choice. In other words, we hypothesize that the small negative effect that people expect nudges to have on autonomy that was found in previous work ([11] but not replicated in Study 1) does not become reality when people are actually nudged, and thus that the nudge conditions do not differ from the control conditions in terms of autonomy and satisfaction.

### Results

#### Descriptives

Over all conditions, 51% of the participants chose the long questionnaire. Similar to Study 1, participants reported relatively high levels of autonomy (*M* = 3.83, *SD* = 0.65), satisfaction (*M* = 4.12, *SD* = 0.57). Autonomy and satisfaction were moderately correlated (*r* = .42, *p* < .01).

#### Randomization check

We conducted an ANOVA with condition as the independent variable and age as the dependent variable and found that randomization was successful (*p* = .450). We also conducted a Chi-squared test and found that sex was successfully randomized across conditions (*p* = .301).

#### Nudge effectiveness

In order to be certain that we investigate the effects of nudges on autonomy and satisfaction after actual choices that were influenced by nudges, we first tested whether the nudge had been effective. We conducted a logistic regression with choice as the dependent variable and condition as the independent variable. While there was no significant difference between the control (45% long version) and social norm nudge condition (48% long version, *p* = .602), the default nudge condition (61% long version) differed significantly from the control condition (*p* = .006). For participants in the default nudge condition, the odds were 1.90 times higher, compared to the control condition, that they would choose the long version of the questionnaire.

#### Autonomy and satisfaction

**Fig 2:**
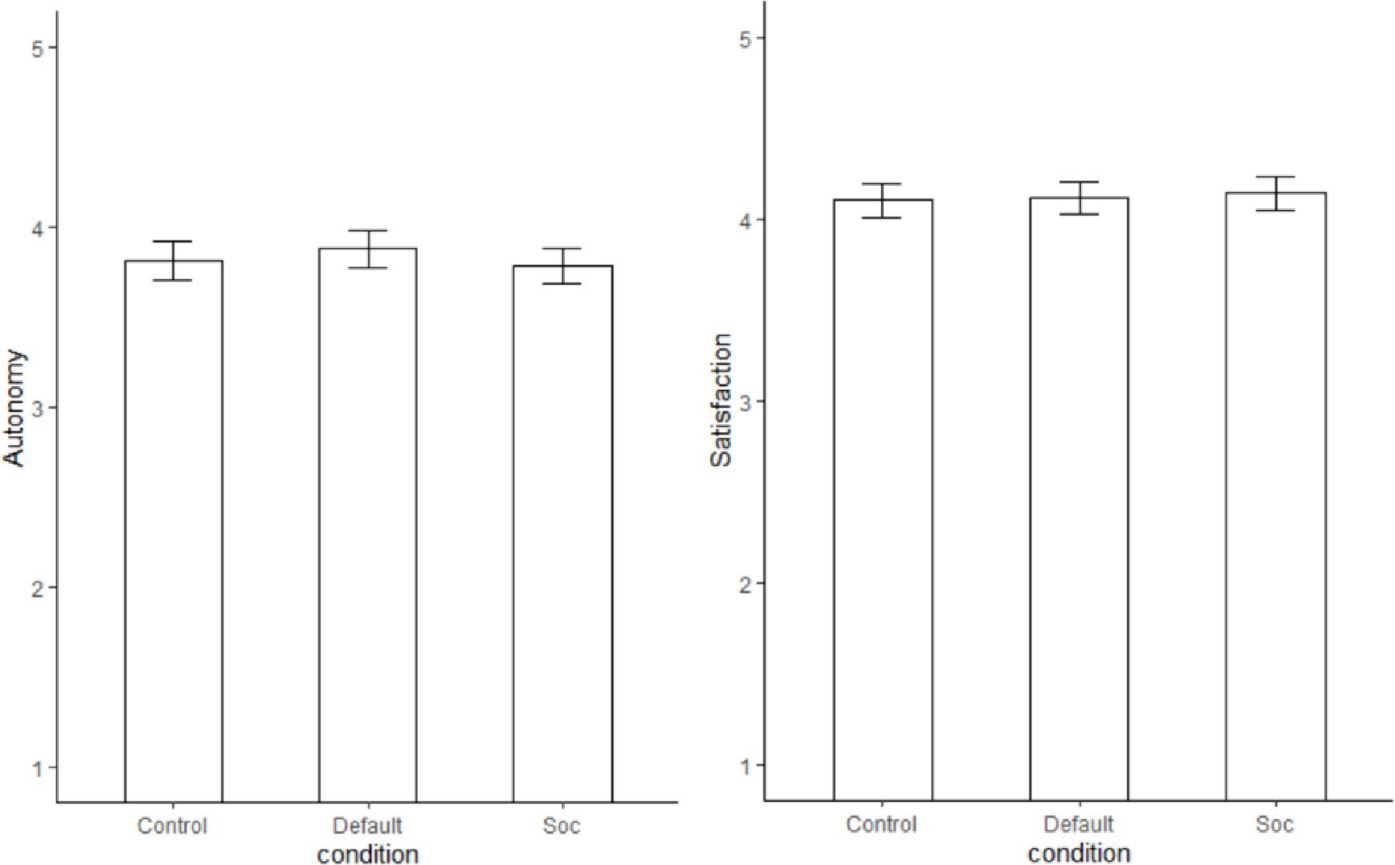
Means and standard errors for autonomy and satisfaction.

We conducted a MANOVA with autonomy and satisfaction as the dependent and condition as the independent variable. The multivariate effect was not significant Wilk’s Λ = .994, *F*(2, 450) = 0.71, p = .585 (See Fig 2 for means and standard errors of all conditions for autonomy and satisfaction).

### Discussion

Similar to Study 1, participants across conditions felt quite autonomous and satisfied about their choice. The default nudge was effective in promoting the long version of the questionnaire, but the social norm nudge was ineffective. However, as hypothesized, the nudge conditions did not differ from the control condition in terms of autonomy or satisfaction with the choice. This indicates that the behavior change that comes with the default nudge has no negative impact on any of the experiences measured in this study. This finding is in contrast to the (marginally) significant difference in participants’ *expectation* of autonomy between the default nudge and the control condition in an earlier (hypothetical) study [11]. As a final test of the effects of nudges on expected vs. actual autonomy, we decided to replicate both Study 1 and Study 2 in one combined design for Study 3. Similar designs as in Study 1 and Study 2 will be used, but the sample size will be increased to reliably determine potentially smaller effects. Additionally, hypothetical and actual nudge data will be collected in one wave, which will minimize influences of sampling and timing.

Furthermore, one could argue that Study 1 and Study 2 were not fully comparable, because in Study 2 participants did not get an explanation of the framing (i.e., default nudge, social norm nudge, control), whereas participants in Study 1 were provided with explanations in the hypothetical scenarios. While we did so on purpose because we wanted to find out whether the experience of autonomy in real-life settings differs from what we expect when we discuss the potential consequences of nudging (implicating that one is aware of what a nudge does), we consider it important to address the factor of explanation once again in Study 3. This will allow us to examine whether and how explanation affects autonomy both in hypothetical and actual settings. To keep the sample size and number of conditions in Study 3 manageable, and because the social proof nudge was not determined effective in Study 2, we decided to incorporate only the default nudge as it is generally viewed as a nudge with relatively high potential violation of autonomy [37].

## Study 3

### Pre-registration

This experiment was pre-registered at https://aspredicted.org/fi6pi.pdf.

### Participants

We recruited 1322 participants (48.3% female, mean age 28.1, *SD* = 9.56 [range 18–73]) through the online service Prolific. Participation took on average 3.25 min and was rewarded with 0.40£. Only Prolific users who had not participated in earlier studies of this research line and were fluent in English were eligible.

### Design and procedure

This online experiment used an asymmetrical three-factor between-subject design, with nudge (default nudge vs control), realism (choice vs hypothetical), and explanation (default nudge with explanation vs. default nudge without explanation vs. control condition without explanation) as the independent variables. This results in six conditions: Choice Nudge Without Explanation (CN-), Choice Nudge with explanation (CN+), Choice Control without explanation (CC-), Hypothetical Nudge without explanation (HN-), Hypothetical Nudge with explanation (HN+), and Hypothetical Control without explanation (HC-; see Table 1).

**Table 1:** Conditions in Study 3.

Realism	Control	Default Nudge	
	Without Explanation	Without Explanation	With Explanation
Choice	CC-	CN-	CN+
Hypothetical	HC-	HN-	HN+

The choice conditions were by and large similar to the default and control conditions in Study 1, with the addition of a choice condition with an explanation (which was presented simultaneously with the nudge). This condition was added to account for “explanation” as a possible confounding factor between Studies 1 and 2. In contrast to Study 2, where participants could immediately make a decision, in Study 3, participants had to wait ten seconds before they could submit their answer (15 seconds for CN+). The hypothetical conditions were also by and large similar to the default nudge and control condition in Study 2, with the addition of a nudge condition without an explanation (HN-). Also, in contrast to Study 2, the control condition did not include an explanation. Finally, the explanation in the hypothetical nudge condition was given simultaneously with the hypothetical question, instead of on the next page as in Study 1.

### Materials

#### Manipulation recollection

Participants in the choice conditions were asked the question, “What was special about the way we asked whether you would like to participate in the long version of the questionnaire?”. They could choose from five answers, stating that either one of the answers was written in bold, or already selected, or that nothing was special, with always only one answer being correct.

#### Pressure

Participants were asked how much pressure they (expected to) experience to select ‘Longer Version‘ as their answer. Participants could answer on a slider ranging from 0 (None at all) to 100 (Extreme Pressure).

### Planned analyses

The aim of Study 3 is to replicate the findings of Study 1 and Study 2 and to do this in one and the same study to minimize the possibility that any differences between hypothetical and choice conditions are due to timing or sampling of the different studies. For both the hypothetical and choice conditions, we seek to compare the individual nudge conditions to their respective control conditions (i.e., to run test within the two condition clusters). Differences between the hypothetical and choice conditions might be due to a variety of reasons, for example, a greater indifference of participants in hypothetical conditions, or that the inability to report a decision in the hypothetical conditions already leads to a decline in autonomy, or that participants in hypothetical conditions are more prone to think from a moral viewpoint and participants in the choice conditions from a pragmatic viewpoint. We therefore find direct comparisons between the hypothetical and choice conditions difficult to interpret and focus on comparisons to the respective control condition within the two reality conditions separately.

The preregistered main hypotheses are that (1) within the choice conditions, autonomy and satisfaction do not differ between the conditions, meaning that neither a nudge, nor its explanation, affect these measures. To test this claim, we will conduct a one-way MANOVA with autonomy and satisfaction as the dependent variables and choice conditions (CC-, CN-, CN+) as the independent variable. However, (2) within the hypothetical conditions, we expect autonomy and satisfaction to differ, with the nudge condition featuring an explanation scoring lowest and the control condition scoring highest on these measures. To test this claim, we will conduct a MANOVA, with autonomy and satisfaction as the dependent variables and the hypothetical conditions (HC-, HN-, HN+) as the independent variable.

### Results

#### Descriptives

Similar to both Study 1 and Study 2, participants reported relatively high (expectations for) autonomy (*M* = 3.88, *SD* = 0.67) and satisfaction (*M* = 4.05, *SD* = 0.62). Autonomy and satisfaction were strongly correlated (*r* = .53, *p* < .01).

#### Randomization check

We conducted an ANOVA with condition as the independent variable and age as the dependent variable and found that randomization of age over the conditions was successful (*p* = .460). A Chi-squared test also showed that sex was successfully randomized across the conditions (*p* = .200).

#### Manipulation check

*Choice conditions*. A logistic regression with Choice (on the nudged question) as the dependent variable and choice conditions as the independent variable with the choice control condition (CC-) as the reference group was conducted to test whether the nudge in the choice conditions was indeed effective. The results showed that neither the choice nudge condition without explanation (CN-) (49.5% long version, *p* = .200) nor including an explanation (CN+) (49.3% long version, *p* = .214) chose significantly different from choice control condition (CC-) (43.4%). We therefore conclude that the nudges were ineffective.

However, when we only included participants who remembered the nudge manipulation, as an indication of paying attention to the manipulation and the questions, the choice nudge condition without explanation (CN-) was effective (CC- 45% long version, CN- 61.6% long version), with percentages being comparable to Study 2 (control: 45%, default nudge: 61%). Testing our main hypotheses about autonomy and satisfaction for this subsample did not yield any different results from the complete sample, therefore all of the analyses below are reported for the complete sample.

#### Aftereffects of nudging: Autonomy and satisfaction

*Choice conditions*. In order to test hypothesis one, we conducted a MANOVA with autonomy and satisfaction as the dependent variables and the choice conditions (CC- / CN-/ CN+) as the independent variable. As expected, the multivariate effect was not significant Wilk’s Λ = .997, *F*(2, 659) = 0.444, p = .780. As we preregistered more specific comparisons, we continued the investigation of effects on autonomy and satisfaction, despite a negative overall effect of condition. However, none of the univariate tests were significant either (all ps > .630). Given the results, we conclude hypothesis one to be supported.

**Fig 3:**
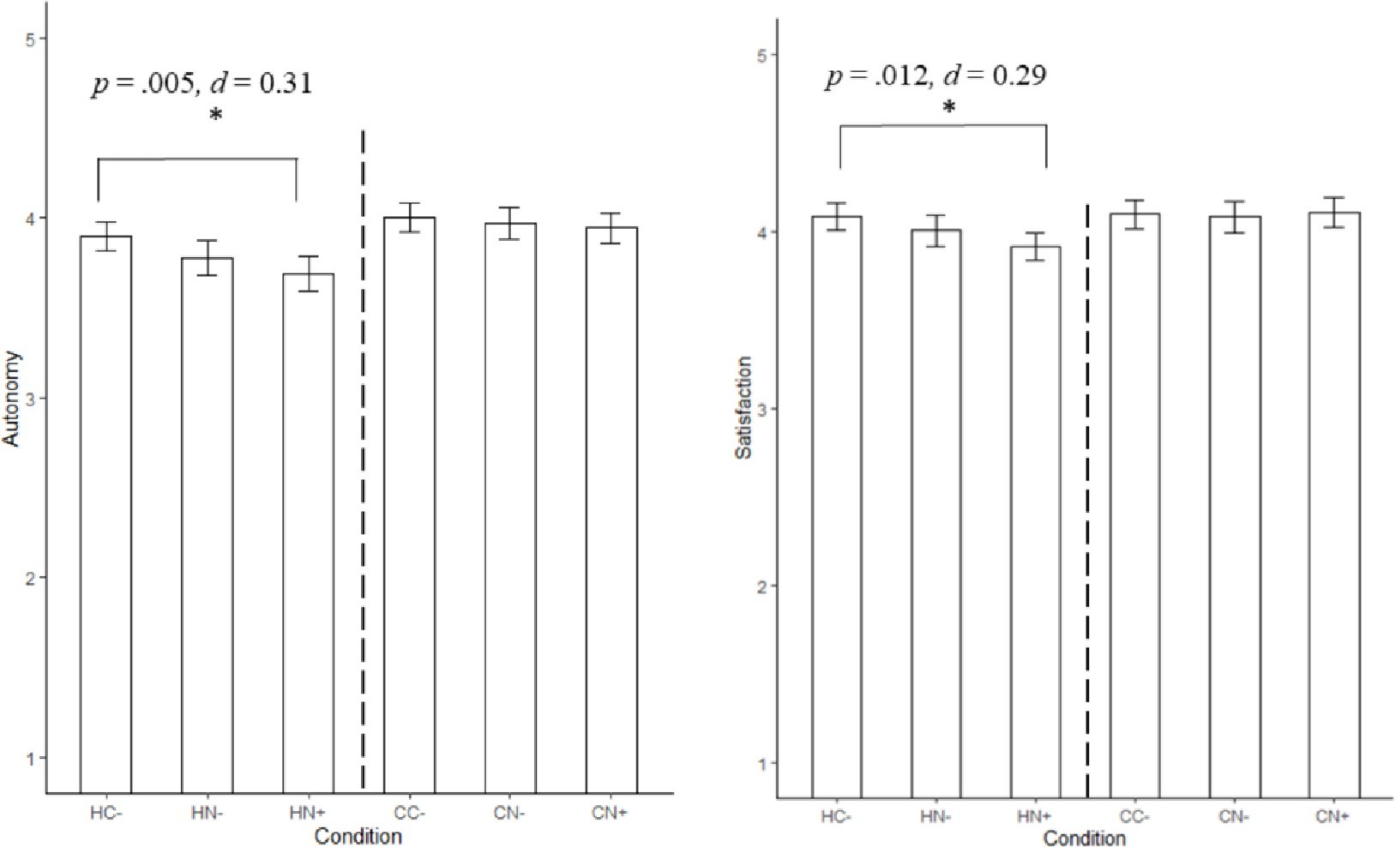
Means and standard errors for autonomy and satisfaction.

*Hypothetical conditions*. To test the second hypothesis, we conducted a MANOVA with autonomy and satisfaction as the dependent and hypothetical conditions (HC- / HN- / HN+) as the independent variable. As expected, the MANOVA resulted in a significant multivariate effect, Wilk’s Λ = .982, F(2, 657) = 3.003, *p* = .018. Univariate tests showed that both autonomy, *F*(2,657) = 4.972, *p* = .007 and satisfaction, *F*(2,657) = 4.109, *p* = .017, differed significantly between the conditions. We subsequently conducted two ANOVAS, with autonomy and satisfaction as the dependent variables, followed by Tukey HSD post-hoc tests, to inspect which conditions differ specifically. As expected, the hypothetical control condition (HC-) scored highest on autonomy (*M* = 3.90, *SD* = 0.60), followed by the hypothetical nudge condition without explanation (HN-) (*M* = 3.78, *SD* = 0.73) and the hypothetical nudge including an explanation (HN+) (*M* = 3.69, *SD* = 0.73). However, only the difference between hypothetical control condition (HC-) and the hypothetical nudge condition including an explanation (HN+) reached significance (HN- vs. HC-: *p* = .154; HN+ vs. HC -: *p* = .005, *d* = 0.31; HN+ vs. HN-: *p* = .395). Similarly, when looking at satisfaction we found that as expected, the hypothetical control condition (HC-) (*M* = 4.08, *SD* = 0.57) scored highest on satisfaction followed by the hypothetical nudge condition without explanation (HN-) (*M* = 4.00, *SD* = 0.66), with the hypothetical nudge condition including an explanation (HN+) scoring the lowest (*M* = 3.92, *SD* = 0.58). Again, only the difference between the hypothetical control condition (HC-) and the hypothetical nudge condition including an explanation (HN+) was significant (HN- vs. HC-: *p* = .350; HN+ vs. HC -: *p* = .012, *d* = 0.29; HN+ vs. HN-: *p* = .293; See Fig 3 for means, standard errors, and significant differences between conditions on autonomy and satisfaction). Given the results, we conclude hypothesis two is partially supported.

#### Correlations between pressure, autonomy and satisfaction

*All conditions*. We calculated correlations between pressure and autonomy, as well as pressure and satisfaction within the four conditions without explanation (HC- / HN-/ CC-/ CN-). As expected, pressure negatively correlated with both autonomy (*r* = -.25, *p* < .001) and satisfaction (*r* = -.31, *p* < .001). We calculated the same correlations within HN+ and CN+, expecting them to be insignificant. However, we again found both pressure and autonomy (*r* = -.27, *p* < .001) and pressure and satisfaction (*r* = -.28, *p* < .001) to be negatively correlated.

### Discussion

Similar to Study 2, we found no negative effect of nudges on autonomy or satisfaction in actual nudge settings. However, negative effects of nudges on autonomy and satisfaction are expected in hypothetical scenarios. In Study 3, these effects for the hypothetical nudge were small but significant, similar to Wachner and colleagues [11]. Participants only felt less autonomous and satisfied after being presented with a hypothetical nudge that included an explanation, while a hypothetical nudge without an explanation did not score significantly lower than the control condition. This suggests that people are not immediately skeptical of the impact of a nudge on autonomy: only when the nudge was explained to them, they feared that the nudge might threaten their autonomy. However, it is uncertain whether this is due to the explanation of how a nudge works or to drawing attention to the presence of a nudge. Taken together, these findings corroborate our expectation that negative expectations of the effects of nudges do not translate into negative effects of nudges on experiences.

Finally, the two choice nudge conditions were not effective in promoting the “Long Version” choice in the initial sample. When only analyzing data from participants who passed the manipulation recollection, the choice nudge without explanation (CN-) was an effective nudge but the choice nudge including an explanation (CN+) was not. Appeasing our earlier concerns, both the sample in which the nudge was effective and the sample in which it was not effective come to the same conclusions in virtually all analyses, suggesting that ineffectiveness of the nudge does not alter our main findings regarding autonomy and satisfaction. Nevertheless, the fact that the default nudge was not effective, and that the transparent nudge condition was less effective than the nudge without explanation, contradicts multiple studies using this specific nudge [28] and studies comparing the effectiveness of transparent and non-transparent nudges [18–20] and remains an important limitation that warrants caution in interpreting our findings.

## General discussion

In the current series of studies, we investigated whether people expect nudges to harm their autonomy and other decision-making experiences and whether nudges harm autonomy when people are actually being nudged. Participants’ *expectations* give us insights into how people think nudging affects autonomy. This is important for when people notice that they are being nudged, or when the use of nudges as a public policy instrument is discussed by laymen or in professional circles, as opinions will be largely derived from expectations of nudges’ influence. Understanding participants’ *actual experiences* after being nudged is crucial for the design of nudges that do not hurt autonomy, as this has been found to lead to a variety of negative effects on well-being [9, 10] and could also negatively affect subsequent related decisions.

Results from a previous study [11], demonstrating small, but significant negative effects of hypothetical nudges on expected autonomy, were not supported in Study 1 but replicated in Study 3, giving credit to the notion that people expect default nudges to harm their autonomy and satisfaction. In Studies 2 and 3, the results showed that when people were actually nudged, autonomy and satisfaction were not affected. These results confirm our hypothesis that nudges are expected to be hurtful to autonomy and satisfaction when people speculate about how they would feel and are asked to imagine themselves in these situations, while these effects do not occur when people are actually nudged. Additionally, when the nudge was hypothetical, participants expected autonomy and satisfaction to be violated only when the nudge was explained to them. Apparently, when the nudge is explained in hypothetical scenarios, people are more concerned about its effects.

Policy makers familiar with the debate regarding the ethics of nudging, and particularly autonomy, may recognize these findings as they suggest that people might be somewhat skeptic of nudges being implemented because of fearing a threat to their autonomy and satisfaction. In the current studies, we did not find an explanation of the nudge to affect autonomy and satisfaction. This is in line with an earlier study in which, similarly, no effect of transparency was found [20].

Despite the concerns people may have regarding their autonomy when they anticipate being nudged, the results for the choice nudge conditions suggest that people do not feel less autonomous and satisfied. It can be argued that these inaccurate expectations are due to participants inexperience with nudges and that people’s predictions will get more accurate as the use of nudges becomes more widespread. However, we expect that pessimistic expectations on the effects of nudges will prevail, due to the already mentioned overestimation effects, such as impact bias [32] and immune neglect [33]. Additionally, as people can be unaware of a nudge being present (and its negative effect on autonomy being absent), they may overlook encounters with nudges that should alter their expectations. We argue that it is therefore important to pay attention to this when designing nudges so as to reduce the expected threat to autonomy.

Overall, our findings are good news for the implementation of nudges. In all conditions, participants expected or experienced relatively high levels of autonomy. Particularly in choice nudge conditions, experienced autonomy was not affected by nudges. In addition, we found in choice conditions that an explanation of the nudge, which makes the decision maker aware of the nudge and the working mechanism, did not negatively affect autonomy and satisfaction, indicating there is no need to refrain from using transparent nudges based on autonomy considerations, as was also previously found [20]. Note that while in the current study the explained nudge was not effective, several studies have found transparent nudges to be just as effective as non-transparent nudges [e.g., 18–20].

The current paradigm that was used to confront participants with (hypothetical) nudges posed only limited stakes to participants. As nudges vary greatly in the heuristics they rely on, their underlying mechanisms, and in the circumstances to which they are applied, a single set of studies prove limited in its generalizability. Future research should replicate the current study with different kinds of nudges, different fields of behavior (e.g., health behavior, saving behavior, etc.), and with choices for which stakes are higher.

All in all, we found that expectations and experiences of autonomy are less of an issue in regards to nudges as might have been expected [7, 8]. Only in hypothetical nudge scenarios did we see negative effects of nudges in one of our studies, but even then autonomy was still expected to be relatively high. The current paper found little to no support for the claim of negative effects of nudges on actual experienced autonomy.

## Supporting information

S1 Appendix (DOCX)

S1 File (DOCX)

S1 Data (ZIP)

## References

[1] Thaler RH, Sunstein, Professor of Law Cass R. Nudge: Improving decisions about health, wealth, and happiness. Yale University Press; 2008.

[2] Van Gestel LC, Kroese FM, De Ridder DTD. Nudging at the checkout counter—A longitudinal study of the effect of a food repositioning nudge on healthy food choice. Psychol Health. 2018;33(6):800–9. doi: 10.1080/08870446.2017.1416116 29252010

[3] Kroese FM, Marchiori DR, de Ridder DTD. Nudging healthy food choices: a field experiment at the train station. J Public Health (Oxf). 2016;38(2):e133–7. 26186924 10.1093/pubmed/fdv096

[4] Madrian BC, Shea DF. The power of suggestion: Inertia in 401(k) participation and savings behavior. Q J Econ. 2001;116(4):1149–87.

[5] Demarque C, Charalambides L, Hilton DJ, Waroquier L. Nudging sustainable consumption: The use of descriptive norms to promote a minority behavior in a realistic online shopping environment. J Environ Psychol. 2015;43:166–74.

[6] Benartzi S, Beshears J, Milkman KL, Sunstein CR, Thaler RH, Shankar M, et al. Should governments invest more in nudging? Psychol Sci. 2017;28(8):1041–55. doi: 10.1177/0956797617702501 28581899 PMC5549818

[7] Bovens L. The ethics of nudge. In: Preference Change. Dordrecht: Springer Netherlands; 2009. p. 207–19.

[8] Hansen PG, Jespersen AM. Nudge and the manipulation of choice: A framework for the responsible use of the nudge approach to behaviour change in public policy. Eur J Risk Regul. 2013;4(1):3–28.

[9] Wei M, Shaffer PA, Young SK, Zakalik RA. Adult attachment, shame, depression, and loneliness: The mediation role of basic psychological needs satisfaction. J Couns Psychol. 2005;52(4):591–601.

[10] Van den Broeck A, Ferris DL, Chang C-H, Rosen CC. A review of self-determination theory’s basic psychological needs at work. J Manage. 2016;42(5):1195–229.

[11] Wachner J, Adriaanse MA, De Ridder DTD. And how would that make you feel? How people expect nudges to influence their sense of autonomy. Front Psychol. 2020;11:607894. doi: 10.3389/fpsyg.2020.607894 33362667 PMC7759476

[12] Fridman I, Hart JL, Yadav KN, Higgins ET. Perspectives on using decision-making nudges in physician-patient communications. PLoS ONE. 2018;13(9): e0202874. 10.1371/journal.pone.0202874 30231040 PMC6145510

[13] Schroeder J, Waytz A, Epley N. Endorsing help for others that you oppose for yourself: mind perception alters the perceived effectiveness of paternalism. J. Exp. Psychol. Gen. 2017. 146, 1106–1125. doi: 10.1037/xge0000320 28557510

[14] Broers VJV, De Breucker C, Van den Broucke S, Luminet O. A systematic review and meta-analysis of the effectiveness of nudging to increase fruit and vegetable choice. Eur J Public Health. 2017;27(5):912–20. doi: 10.1093/eurpub/ckx085 28655176

[15] Arno A, Thomas S. The efficacy of nudge theory strategies in influencing adult dietary behaviour: a systematic review and meta-analysis. BMC Public Health [Internet]. 2016;16(1). Available from: 10.1186/s12889-016-3272-x 27475752 PMC4967524

[16] Hummel D, Maedche A. How effective is nudging? A quantitative review on the effect sizes and limits of empirical nudging studies. J Behav Exp Econ. 2019;80:47–58.

[17] Vugts A, Verweij M, de Vet E, Van Den Hoven M. Which conceptualisations of autonomy are addressed in the ethical discussion on nudging?. European Health Psychologist. 2016;18:1066.

[18] Bruns H, Kantorowicz-Reznichenko E, Klement K, Luistro Jonsson M, Rahali B. Can nudges be transparent and yet effective? J Econ Psychol. 2018;65:41–59.

[19] Paunov Y, Wänke M, Vogel T. Ethical defaults: which transparency components can increase the effectiveness of default nudges? Soc Influ. 2019;14(3–4):104–16.

[20] Wachner J, Adriaanse M, De Ridder D. The influence of nudge transparency on the experience of autonomy. Compr Results Soc Psychol. 2020;1–15.

[21] Wilkinson TM. Nudging and manipulation. Polit Stud. 2013;61(2):341–55.

[22] Ryan RM, Deci EL. Self-determination theory and the facilitation of intrinsic motivation, social development, and well-being. Am Psychol. 2000;55(1):68–78. doi: 10.1037//0003-066x.55.1.68 11392867

[23] Sheldon KM, Ryan R, Reis HT. What makes for a good day? Competence and autonomy in the day and in the person. Pers Soc Psychol Bull. 1996;22(12):1270–9.

[24] Cordeiro P, Paixão P, Lens W, Lacante M, Sheldon K. Factor structure and dimensionality of the balanced measure of psychological needs among Portuguese high school students. Relations to well-being and ill-being. Learn Individ Differ. 2016;47:51–60.

[25] Fotiadis A, Abdulrahman K, Spyridou A. The mediating roles of psychological autonomy, competence and relatedness on work-life balance and well-being. Front Psychol. 2019;10:1267. doi: 10.3389/fpsyg.2019.01267 31191420 PMC6549400

[26] Wirtz D, Kruger J, Napa Scollon C, Diener E. What to do on spring break? The role of predicted, on-line, and remembered experience in future choice: The role of predicted, on-line, and remembered experience in future choice. Psychol Sci. 2003;14(5):520–4. doi: 10.1111/1467-9280.03455 12930487

[27] Ariely D, Norton MI. How actions create–not just reveal–preferences. Trends in cognitive sciences. 2008;12(1):13–16. doi: 10.1016/j.tics.2007.10.008 18063405

[28] Van Rookhuijzen M, De Vet E, Adriaanse MA. The effects of nudges: One shot only? Exploring the temporal spillover effects of a default nudge. Front. Psychol. 2021. doi: 10.3389/fpsyg.2021.683262 PMC8474638 34589018

[29] Dunn EW, Forrin ND, Ashton-James CE. On the excessive rationality of the emotional imagination: A two-systems account of affective forecasts and experiences. In: Handbook of Imagination and Mental Simulation. Routledge; 2015.

[30] Gilbert DT, Wilson TD. Miswanting: Some problems in the forecasting of future affective states. In: Lichtenstein S, Slovic P, editors. The Construction of Preference. Cambridge: Cambridge University Press; 2009. p. 550–64.

[31] Buehler R, McFarland C. Intensity bias in affective forecasting: The role of temporal focus. Pers Soc Psychol Bull. 2001;27(11):1480–93.

[32] Wilson TD, Gilbert DT. The impact bias is alive and well. J Pers Soc Psychol. 2013;105(5):740–8. doi: 10.1037/a0032662 24219785

[33] Wilson TD, Gilbert DT. Affective forecasting: Knowing what to want. Curr Dir Psychol Sci. 2005;14(3):131–4.

[34] Vlachopoulos SP, Michailidou S. Development and initial validation of a measure of autonomy, competence, and relatedness in exercise: The basic psychological needs in exercise scale. Meas Phys Educ Exerc Sci. 2006;10(3):179–201.

[35] Brehaut JC, O’Connor AM, Wood TJ, Hack TF, Siminoff L, Gordon E, et al. Validation of a decision regret scale. Med Decis Making. 2003;23(4):281–92. doi: 10.1177/0272989X03256005 12926578

[36] Rosenberg BD, Siegel JT. A 50-year review of psychological reactance theory: Do not read this article. Motiv Sci. 2018;4(4):281–300.

[37] Jung JY., & Mellers BA. American attitudes toward nudges. Judgment and Decision Making. 2016. 11(1):62–74.

