# Supplementary material for: The effect of nudges on autonomy in hypothetical and real life settings
Source: PLoS One. 2021 Aug 24;16(8):e0256124. doi: 10.1371/journal.pone.0256124 (PMC8384220; doi:10.1371/journal.pone.0256124)
Supplement: S1 Appendix — (DOCX) [file pone.0256124.s001.docx]

S1 Appendix

Autonomy Questionnaire

My choice is highly compatible with my goals and interests.

I feel very strongly that my choice perfectly fits my taste.

I feel that my choice is definitely an expression of myself.

I feel very strongly that I had the opportunity to have influence on my choice.

(Strongly disagree, disagree, neither agree nor disagree, agree, strongly agree)

Satisfaction with Choice Questionnaire

My decision is sound

I am comfortable with my decision

My decision is the right one for my situation

I am satisfied with my decision

It was difficult to make a choice*

(strongly disagree, disagree, neither agree nor disagree, agree, strongly agree)
