# Supplementary material for: The effect of nudges on autonomy in hypothetical and real life settings
Source: PLoS One. 2021 Aug 24;16(8):e0256124. doi: 10.1371/journal.pone.0256124 (PMC8384220; doi:10.1371/journal.pone.0256124)
Supplement: S1 File — (DOCX) [file pone.0256124.s002.docx]

Supplemental Materials Study 1

**Hypotheses**

We hypothesized experienced pressure and feeling commanded to be higher in each of the nudge conditions, compared to the control condition.

**Methods**

*Decision-making competence questionnaire.* Participants’ expected competence was measured with a questionnaire consisting of six statements (e.g., “I think I am pretty good at making these kinds of decisions.”), which were rated on five-point scales (“strongly disagree” to “strongly agree”; See Appendix). The five scores were averaged to one competence score with acceptable reliability (Cronbach’s α = .78).

*Pressure.* Participants were asked in all three studies how much pressure they (expected to) experience to select ‘Longer Version‘ as their answer. Participants could answer on a slider ranging from 0 (None at all) to 100 (Extreme Pressure).

*Commandingness.* Participants rated how commanding they thought the question to be (“Would you describe the question about the length of the questionnaire to be demanding in a dominant way?”) on a slider ranging from 0 (Not dominant at all) to 100 (Very dominant).

**Results**

Table 1

*Means, standard deviations, and correlations with confidence intervals*

| Variable | *M* | *SD* | 1 | 2 | 3 | 4 | 5 | 6 |
| --- | --- | --- | --- | --- | --- | --- | --- | --- |
|  |  |  |  |  |  |  |  |  |
| 1. Autonomy | 3.85 | 0.67 |  |  |  |  |  |  |
|  |  |  |  |  |  |  |  |  |
| 2. Satisfaction | 4.02 | 0.61 | .59** |  |  |  |  |  |
|  |  |  | [.53, .65] |  |  |  |  |  |
|  |  |  |  |  |  |  |  |  |
| 3. Competence | 3.70 | 0.60 | .57** | .58** |  |  |  |  |
|  |  |  | [.50, .63] | [.51, .63] |  |  |  |  |
|  |  |  |  |  |  |  |  |  |
| 4. Pressure | 33.99 | 29.82 | -.24** | -.28** | -.24** |  |  |  |
|  |  |  | [-.33, -.15] | [-.37, -.20] | [-.33, -.15] |  |  |  |
|  |  |  |  |  |  |  |  |  |
| 5. Dominance | 36.10 | 27.29 | -.11* | -.22** | -.10* | .46** |  |  |
|  |  |  | [-.20, -.02] | [-.31, -.13] | [-.19, -.01] | [.39, .53] |  |  |
|  |  |  |  |  |  |  |  |  |
| 6. Age | 29.58 | 10.48 | .01 | .10* | .14** | -.08 | -.12* |  |
|  |  |  | [-.08, .10] | [.01, .19] | [.05, .23] | [-.17, .01] | [-.21, -.03] |  |
|  |  |  |  |  |  |  |  |  |
| 7. Sex | 0.47 | 0.51 | .04 | .09 | .04 | .05 | -.06 | .13** |
|  |  |  | [-.05, .13] | [-.01, .18] | [-.05, .13] | [-.04, .15] | [-.15, .04] | [.04, .22] |
|  |  |  |  |  |  |  |  |  |

*Note.* Correlation Matrix for descriptive data from Study 1. *M* and *SD* are used to represent mean and standard deviation, respectively. * indicates *p* < .05. ** indicates *p* < .01.

**Competence**

We conducted an ANOVA with competence as the dependent variable and condition as the independent variable. No significant effect of condition on competence was found, *F*(2,448) = 2.00, *p* = .140.

**Pressure and Commandingness**

We conducted a MANOVA with pressure and commandingness as the dependent, and condition as the independent variable. The multivariate effect was significant Wilk’s Λ = .943, *F*(2, 448) = 6.70, p < .001. The univariate tests show no significant effect on pressure *F*(2, 448) = 2.73, p = .066, but show a significant effect on commandingess *F*(2, 448) = 12.8, p < .001.

A post-hoc Tukey HSD test on pressure showed a marginal difference between the social norm nudge (*M* = 37.59, *SD* = 29.54) and control condition (*M* = 29.67, *SD* = 30.41, *p* = .055). No significant effects were found between the default nudge (*M* = 34.69, *SD* = 29.17) and control condition (*p* = .311) and the default and social norm condition (*p* = .674).

A second post-hoc Tukey HSD test on commandingness showed that the control condition (*M* = 27.15, *SD* = 25.51) scored significantly lower than both the default nudge condition (*M* = 41.35, *SD* = 28.05, *p* < .001, *d* = -.53) and the social norm nudge condition (*M* = 39.76, *SD* = 26.19, *p* < .001, *d* = -.49). The default and social norm condition did not differ significantly (*p* = .860).

**Discussion**

We found no support for our hypotheses that participants in the default nudge condition expect to feel less competent than participants in the control condition, and that participants in the social norm nudge condition expect to feel more competent compared to the control condition, as we found no differences in expected competence between any of the conditions.

Our hypothesis that the nudge conditions score higher on pressure than the control conditions was not supported. However, similar to the findings for autonomy from the main article, the differences that we found, while not significant, showed a clear trend in the expected direction. The same hypothesis for commandingness was partially supported, as the both nudge scored higher than the control condition.

Supplemental Materials Study 2

**Materials**

The materials were mostly the same, except the secondary measure commandingness was replaced by the secondary measures of care and doubt.

*Doubt.*  Doubt was measured with one question: ‘Did you doubt that your answer would alter the survey’s length?’. Participants answered on a slider ranging from 0 (‘Not at all’) to 100 (‘Very much’).

*Care.* Care was also measured with one question: “How carefully did you decide? “ (referring to the question concerning the length of the questionnaire). Participants answered on a scale from 0 to 100 with the same labels as the doubt measure.

| Variable | *M* | *SD* | 1 | 2 | 3 | 4 | 5 | 6 | 7 | 8 |
| --- | --- | --- | --- | --- | --- | --- | --- | --- | --- | --- |
|  |  |  |  |  |  |  |  |  |  |  |
| 1. Choice | 0.51 | 0.50 |  |  |  |  |  |  |  |  |
|  |  |  |  |  |  |  |  |  |  |  |
| 2. Autonomy | 3.83 | 0.65 | .12* |  |  |  |  |  |  |  |
|  |  |  | [.03, .21] |  |  |  |  |  |  |  |
|  |  |  |  |  |  |  |  |  |  |  |
| 3. Satisfaction | 4.12 | 0.57 | .13** | .42** |  |  |  |  |  |  |
|  |  |  | [.04, .22] | [.34, .49] |  |  |  |  |  |  |
|  |  |  |  |  |  |  |  |  |  |  |
| 4. Competence | 3.67 | 0.57 | .11* | .52** | .56** |  |  |  |  |  |
|  |  |  | [.02, .20] | [.45, .58] | [.50, .62] |  |  |  |  |  |
|  |  |  |  |  |  |  |  |  |  |  |
| 5. Pressure | 32.08 | 28.56 | -.21** | -.26** | -.29** | -.25** |  |  |  |  |
|  |  |  | [-.29, -.12] | [-.34, -.17] | [-.37, -.21] | [-.34, -.16] |  |  |  |  |
|  |  |  |  |  |  |  |  |  |  |  |
| 6. Care | 63.66 | 26.71 | .00 | .21** | .16** | .15** | .04 |  |  |  |
|  |  |  | [-.09, .09] | [.12, .30] | [.07, .25] | [.06, .24] | [-.06, .13] |  |  |  |
|  |  |  |  |  |  |  |  |  |  |  |
| 7. Doubt | 37.54 | 32.02 | -.21** | -.11* | -.18** | -.08 | .32** | .08 |  |  |
|  |  |  | [-.29, -.12] | [-.20, -.02] | [-.26, -.08] | [-.17, .02] | [.24, .40] | [-.01, .17] |  |  |
|  |  |  |  |  |  |  |  |  |  |  |
| 8. Age | 29.17 | 9.58 | .19** | .09 | .08 | .14** | -.12* | .05 | -.08 |  |
|  |  |  | [.10, .28] | [-.00, .18] | [-.01, .17] | [.05, .23] | [-.21, -.03] | [-.05, .14] | [-.17, .02] |  |
|  |  |  |  |  |  |  |  |  |  |  |
| 9. Sex | 0.50 | 0.50 | .17** | -.03 | .07 | .09 | .00 | -.04 | -.05 | .08 |
|  |  |  | [.07, .25] | [-.12, .06] | [-.02, .16] | [-.00, .18] | [-.09, .10] | [-.13, .05] | [-.14, .05] | [-.01, .17] |
|  |  |  |  |  |  |  |  |  |  |  |

Table 2

*Means, standard deviations, and correlations with confidence intervals*

*Note.* Correlation Matrix for descriptive data from Study 2. *M* and *SD* are used to represent mean and standard deviation, respectively.

**Results**

**Condition and Choice on Autonomy and Satisfaction**

We investigated whether condition and choice had an interactive effect on autonomy and satisfaction. We conducted a MANOVA with autonomy and satisfaction as the dependent, and condition and choice as the independent variables. We found no univariate multivariate effect of condition Wilk’s Λ = .994, *F*(4, 892) = 0.716, p = .581, but we did find a significant effect of choice Wilk’s Λ = .979, *F*(2, 446) = 4.863, p = .009. Finally, the interaction term of condition and choice had no significant effect Wilk’s Λ = .984, *F*(4, 892) = 1.786, p = .130.

**Competence**

We conducted an ANOVA with competence as the dependent variable and condition as the independent variable, which resulted in no effect of condition on competence *F*(2,450) = 0.05, *p* = .960.

**Pressure, Care, and Doubt**

Given that pressure and doubt were correlated, we analyzed both in one MANOVA, with condition as the independent variable. The multivariate effect was not significant Wilk’s Λ = .993, *F*(2, 450) = 0.769, p = .546.

We also conducted an ANOVA with care as the dependent variable and condition as the independent variable. The effect was not significant *F*(2,450) = 0.21, *p* = .810.

**Supplemental Materials Study 3**

**Materials**

*Acceptance.* Acceptance was measured by asking participants in the choice nudge conditions how acceptable it was for the researchers to pre-select the answer ’Longer Version’ in order to promote this choice. Participants in the choice nudge condition without explanation, who had not yet received an explanation of the nudge, were presented with it next to the acceptance question. Acceptance was measured on a slider ranging from 0 (“Very Unacceptable”) to 100 (“Fully Acceptable”). This measure was exploratory and not pre-registered. Analyses on acceptance can be found in the supplemental materials.

**Hypotheses**

Besides the planned contrasts, hypothesis three (3) predicts that pressure will correlate negatively with both autonomy and satisfaction across all conditions without an explanation, while there will be no correlations in conditions where the nudge is explained. Such a finding would support the idea that explaining the nudge would give the experienced pressure context and not be perceived as autonomy threatening, leading to no harm to one’s satisfaction. This will be tested by calculating four correlations, two for autonomy and pressure, two for satisfaction and pressure, one for conditions without explanation, and one for conditions with an explanation.

**Results**

Table 3

*Means, standard deviations, and correlations with confidence intervals*

| Variable | *M* | *SD* | 1 | 2 | 3 | 4 | 5 |
| --- | --- | --- | --- | --- | --- | --- | --- |
|  |  |  |  |  |  |  |  |
| 1. Autonomy | 3.88 | 0.67 |  |  |  |  |  |
|  |  |  |  |  |  |  |  |
| 2. Satisfaction | 4.05 | 0.62 | .53** |  |  |  |  |
|  |  |  | [.49, .56] |  |  |  |  |
|  |  |  |  |  |  |  |  |
| 3. Competence | 3.70 | 0.59 | .53** | .58** |  |  |  |
|  |  |  | [.49, .56] | [.54, .62] |  |  |  |
|  |  |  |  |  |  |  |  |
| 4. Pressure | 31.19 | 28.68 | -.26** | -.30** | -.28** |  |  |
|  |  |  | [-.31, -.21] | [-.35, -.25] | [-.33, -.23] |  |  |
|  |  |  |  |  |  |  |  |
| 5. Age | 28.11 | 9.56 | .04 | .08** | .11** | -.04 |  |
|  |  |  | [-.01, .09] | [.03, .13] | [.06, .16] | [-.10, .01] |  |
|  |  |  |  |  |  |  |  |
| 6. Sex | 0.48 | 0.50 | .04 | .09** | .11** | -.00 | .16** |
|  |  |  | [-.02, .09] | [.04, .14] | [.06, .17] | [-.05, .05] | [.11, .21] |
|  |  |  |  |  |  |  |  |

*Note.* Correlation Matrix of the initial data from Study 3. *M* and *SD* are used to represent mean and standard deviation, respectively. Values in square brackets indicate the 95% confidence interval for each correlation. * indicates *p* < .05. ** indicates *p* < .01.

**Condition and Choice on Autonomy and Satisfaction**

*Choice Conditions.* We investigated whether condition and choice had an interactive effect on autonomy and satisfaction. We conducted a MANOVA with autonomy and satisfaction as the dependent, and condition and choice as the independent variables. We found no univariate multivariate effect of condition Wilk’s Λ = .997, *F*(4, 1310) = 0.450, p = .770, but we did find a significant effect of choice Wilk’s Λ = .952, *F*(2, 655) = 16.45, p < .001. Finally, the interaction term of condition and choice had no significant effect Wilk’s Λ = .990, *F*(4, 1310) = 1.730, p = .140.

**Aftereffects of Nudging: Competence**

*Choice Conditions.* In order to test hypothesis one, we conducted an ANOVA with competence as the dependent variables and the choice condition (CC- / CN-/ CN+) as the independent variable. No effect of condition on competence was found *F*(2/659) = 1.04, *p* = .360).

Similarly, we conducted an ANOVA with hypothetical condition (HC-, HN-, HN+) as the independent variable. Similarly, we found no effects on hypothetical condition on competence *F*(2/657) = 1.58, *p* = .210).

**Nudge Explanation and Realism on Pressure**

*Nudge conditions.* We decided to also investigate whether an explanation leads to higher experienced pressure. Therefore, we conducted an ANOVA on data of only the nudge conditions (i.e. without hypothetical control condition and choice control condition). Pressure was set as the dependent variable, explanation (with explanation/without explanation), realism (hypothetical/choice), and their interaction term was set as independent variables. No effect of explanation on pressure was found *F*(1/878) = 0.33, *p* = .570. Realism, however, had a significant effect on pressure *F*(1/878) = 16.42, *p* < .001, with hypothetical conditions (*M* = 35.47, *SD* = 29.10) scoring significantly higher on pressure than the choice conditions (*M* = 27.74, *SD* = 27.57). The interaction term was not significant *F*(1/878) = 1.23, *p* = .270.

**Acceptability**

We also investigated acceptance as an outcome variable, to investigate how participants experienced the different nudges. We therefore conducted two Welch two sample t-tests to compare how acceptable the participants rated the nudge in the two hypothetical nudge conditions and in the two choice nudge conditions. The comparison between the two hypothetical conditions showed that HN- (*M* = 47.74, *SD* = 32.28) rated the nudge as significantly less acceptable than HN+ (*M* = 54.73, *SD* = 33.39), *t*(437) = -2.2, *p* = .026; *d* = 0.21. The same comparison between the choice nudge conditions revealed a similar difference, with CN- (*M* = 56.37, *SD* = 31.39) rating the nudge as significantly less acceptable than CN+ (*M* = 63.69, *SD* = 29.02), *t*(434) = -2.5, *p* = .011^[[1]](#footnote-1)^; *d* = 0.24. We then conducted an ANOVA with realism, explanation, and their interaction term as the independent variable, and acceptance as the dependent variable. The ANOVA showed a significant effect for realism, *F*(1,878) = 17.128, *p* < .001, *d* = 0.28, with the hypothetical conditions (*M* = 51.20, *SD* = 32.98) scoring significantly lower on acceptance than the choice conditions (*M* = 60.07, *SD* = 30.40). Explanation did also have a significant effect on acceptance *F*(1,878) = 11.648, *p* < .001, *d* = 0.23, with conditions without an explanation (*M* = 52.01, *SD* = 32.10) being less acceptant of the nudge than conditions with an explanation (*M* = 59.26, *SD* = 31.54). There was not effect of the interaction term on acceptance (*p* = .937).

**Discussion**

It was found that pressure correlated negatively with expected autonomy and satisfaction within conditions without an explanation, as predicted in hypothesis 3. Contrary to hypothesis 4, these correlations are equally strong in conditions with explanations. This suggests that pressure equally affects autonomy and satisfaction when the nudge is explained as compared to when the nudge is not explained.

Acceptance was, however, higher in conditions with an explanation. Additionally, hypothetical conditions scored lower on acceptance than the choice conditions. The first finding suggests that while an explanation does not lead to a weaker relationship between pressure and autonomy, it still leads to a higher general acceptance of the nudge. The finding that in hypothetical scenarios nudges are seen as less acceptable, in combination with negative effects of nudges on autonomy in hypothetical scenarios but not choice scenarios, suggests that people are generally more critical of nudges when they are not asked to make a choice, but rather evaluate the use of nudges.

**Appendix**

Decision-Making Competence Questionnaire

I think I am pretty good at making these kinds of decisions.

I think I did pretty well at making this decision, compared to other people.

After making this decision, I feel competent.

I am satisfied with my performance at this decision.

I was pretty skilled at making this decision.

This was an activity that I couldn't do very well.*

(strongly disagree, disagree, neither agree nor disagree, agree, strongly agree)

Statements with an asterisk are mirrored questions.

1. This is the only instance of a difference in statistical significance between the initial sample and the sample only containing participants who passed the manipulation recollection. With the latter sample, *p* = .061). [↑](#footnote-ref-1)
